# A Case of Laryngeal Phthisis, in Which the Windpipe Was Opened on Account of Dyspnœa

**Published:** 1884-12

**Authors:** Christopher Elliott

**Affiliations:** Physician to the Bristol Hospital for Sick Children


### Clinical Records.

---

A CASE OF LARYNGEAL PHTHISIS, IN WHICH THE WINDPIPE WAS OPENED ON ACCOUNT OF DYSPNŒA. By CHRISTOPHER ELLIOTT, M.D., Physician to the Bristol Hospital for Sick Children.

E. R., æt. 6, the child of a dock labourer, was admitted into the Children's Hospital, under my care, with the following history: For the last six months she had been gradually losing her voice, and her breathing had become bad. She had no cough in the daytime, but at night would get attacks of dyspnœa and coughing. She never had suffered from croup, but three years ago had a sharp attack of broncho-pneumonia. There was no history of syphilis; both her parents and their other children were all healthy and living, but a maternal aunt had died of consumption. The day after admission it was noticed that the patient had some difficulty in swallowing food, especially liquids. She had one or two enlarged glands in the upper part of the neck on both sides, but there was no swelling in the pharynx, and no tenderness or deformity over the upper cervical vertebræ. As I was away for a holiday when my patient was admitted into the hospital, I did not see her until September 7th. Her respiration was then noisy and stridulous. She had no cough, and could not speak above a whisper. The head was thrown backwards, and kept fixed in this position ; the sterno-mastoid muscles on both sides were tensely contracted, and the lower end of the sternum was drawn in deeply at each inspiration. Over the third rib, a little below the right clavicle, was a hard osseous nodule. Physical examination of the chest showed that there was very little air entering the lungs, especially the apices, and some slight dulness in front over the left apex. All attempts to ascertain the condition of the larynx with the laryngoscope failed, in consequence of the dyspnœa and spasm caused by the introduction of the instrument, and the collections of mucus and saliva at the back of the throat. In the night of September 12th the patient had an unusually severe attack of dyspnœa, and the next morning, after a consultation with the other members of the staff, my colleague, Mr. Norton, performed laryngo-tracheotomy. The operation was skilfully done, and gave immediate relief. The stridor and dyspnœa at once disappeared, and afterwards the patient was able to take food much better, only a little fluid now and then coming through the tube. For a fortnight the patient seemed to improve and pick up a little bit ; but after this she began rapidly to waste and lose strength. The dulness over the left lung increased, and the right lung also became dull. The evening temperature rose to above 103°, night sweats set in, and the patient died on October 14th. Five days before death took place it was noticed that respiration was carried on partly through the nose and mouth, and partly through the tube ; and when the tube was completely blocked, the patient could breathe without distress. The tube was therefore removed, and the opening allowed to close ; but the voice could not be raised above a low whisper.

*Post Mortem.*—Six hours after death, the tongue, larynx, œsophagus, lungs and heart were all removed together for examination.

*Larynx.*—No trace of vocal cords, either true or false. The whole of the laryngeal mucous membrane was pale and thickened, and had patches of ulceration. The epiglottis was a little thickened, but not ulcerated. On the right side of the larynx, near the posterior part and above the position of the right vocal cord, a small abscess cavity was found, which had burst, and was probably the size of a large pea when full, and had been most likely the cause of the attacks of suffocation the patient had suffered from. Below the thyroid cartilage, the trachea seemed healthy, and there was no sign of irritation where the tube had been worn.

*Œsophagus* was quite healthy.

*Lungs.*—The left lung was moderately adherent to the chest wall over most of its surface. It was full of miliary tubercles, about the size of a pin's head, and scarcely crepitant in any part. Right lung: the upper lobe was solid, and full of same tubercles. There were no cavities in either lung.

The bronchial glands were all considerably enlarged, but not cheesy. The *heart* was normal. The liver and kidneys were free from tubercle. Both *recurrent laryngeal nerves* were dissected out in their entire course, and found to be embedded throughout a large part of their course in enlarged glands. The nodule felt during life over the third rib turned out to be an old fracture of this rib, which had badly united, and was joined on to the one above it.

*Remarks.*—The necessity of opening the trachea in laryngeal phthisis very rarely occurs, according to Morell

Mackenzie. In his book on *Diseases of the Throat and Nose*, he gives a list of 500 cases, and in three only was there such extreme dyspnœa as required to be relieved by tracheotomy. Though the operation prolonged my patient's life only for a month, yet it enabled her to pass her last days in comparative ease and quietness, without being liable to distressing suffocative attacks at night, or whenever she partook of food. It is remarkable that, notwithstanding the complete destruction and disappearance of the vocal cords, this patient had no difficulty in making known her wants, as she was always able to speak in low whisper; and I was not prepared to find such a condition of parts in the larynx as the autopsy revealed. The finding of the abscess cavity in the posterior part of the larynx, above the site of the right vocal cord, accounts, I think, for the dyspnœa she suffered from, and its bursting for the disappearance of this symptom before death.

---

